# The BMP Receptor 2 in Pulmonary Arterial Hypertension: When and Where the Animal Model Matches the Patient

**DOI:** 10.3390/cells9061422

**Published:** 2020-06-08

**Authors:** Chris Happé, Kondababu Kurakula, Xiao-Qing Sun, Denielli da Silva Goncalves Bos, Nina Rol, Christophe Guignabert, Ly Tu, Ingrid Schalij, Karien C. Wiesmeijer, Olga Tura-Ceide, Anton Vonk Noordegraaf, Frances S. de Man, Harm Jan Bogaard, Marie-José Goumans

**Affiliations:** 1 Amsterdam UMC, Vrije Universiteit Amsterdam, Department of Pulmonology, Amsterdam Cardiovascular Sciences, 1081 HV Amsterdam, The Netherlands; c.happe@amsterdamumc.nl (C.H.); x.sun@amsterdamumc.nl (X.-Q.S.); dsgbos@gmail.com (D.d.S.G.B.); n.rol@amsterdamumc.nl (N.R.); i.schalij@amsterdamumc.nl (I.S.); a.vonk@amsterdamumc.nl (A.V.N.); fs.deman@amsterdamumc.nl (F.S.d.M.); hj.bogaard@amsterdamumc.nl (H.J.B.); 2 Laboratory for Cardiovascular Cell Biology, Department of Cell and Chemical Biology, Leiden University Medical Center, 2300 RC Leiden, The Netherlands; k.b.kurakula@lumc.nl (K.K.); C.C.Wiesmeijer@lumc.nl (K.C.W.); 3 INSERM UMR_S 999, Hôpital Marie Lannelongue, 92350 Le Plessis-Robinson, France; christophe.guignabert@inserm.fr (C.G.); lyieng@gmail.com (L.T.); 4 Université Paris-Saclay, School of Medicine, 94270 Le Kremlin-Bicêtre, France; 5 Department of Pulmonary Medicine, Hospital Clínic-Institut d’Investigacions Biomèdiques August Pi I Sunyer (IDIBAPS), University of Barcelona, 08036 Barcelona, Spain; TURA@clinic.cat; 6 Biomedical Research Networking center on Respiratory diseases (CIBERES), 28029 Madrid, Spain; 7 Department of Pulmonary Medicine, Dr. Josep Trueta University Hospital de Girona, Santa Caterina Hospital de Salt and the Girona Biomedical Research Institut (IDIBGI), 17190 Girona, Catalonia, Spain

**Keywords:** pulmonary arterial hypertension, BMPR2, BMP and TGF-β signaling, animal models of pulmonary hypertension

## Abstract

**Background:** Mutations in bone morphogenetic protein receptor type II (BMPR2) are leading to the development of hereditary pulmonary arterial hypertension (PAH). In non-hereditary forms of PAH, perturbations in the transforming growth factor-β (TGF-β)/BMP-axis are believed to cause deficient BMPR2 signaling by changes in receptor expression, the activity of the receptor and/or downstream signaling. To date, BMPR2 expression and its activity in the lungs of patients with non-hereditary PAH is poorly characterized. In recent decades, different animal models have been used to understand the role of BMPR2 signaling in PAH pathophysiology. Specifically, the monocrotaline (MCT) and Sugen–Hypoxia (SuHx) models are extensively used in interventional studies to examine if restoring BMPR2 signaling results in PAH disease reversal. While PAH is assumed to develop in patients over months or years, pulmonary hypertension in experimental animal models develops in days or weeks. It is therefore likely that modifications in BMP and TGF-β signaling in these models do not fully recapitulate those in patients. In order to determine the translational potential of the MCT and SuHx models, we analyzed the BMPR2 expression and activity in the lungs of rats with experimentally induced PAH and compared this to the BMPR2 expression and activity in the lungs of PAH patients. **Methods:** the BMPR2 expression was analyzed by Western blot analysis and immunofluorescence (IF) microscopy to determine the quantity and localization of the receptor in the lung tissue from normal control subjects and patients with hereditary or idiopathic PAH, as well as in the lungs of control rats and rats with MCT or SuHx-induced PAH. The activation of the BMP pathway was analyzed by determining the level and localization of phosphorylated Smad1/5/8 (pSmad 1/5/8), a downstream mediator of canonical BMPR2 signaling. **Results:** While BMPR2 and pSmad 1/5/8 expression levels were unaltered in whole lung lysates/homogenates from patients with hereditary and idiopathic PAH, IF analysis showed that BMPR2 and pSmad 1/5/8 levels were markedly decreased in the pulmonary vessels of both PAH patient groups. Whole lung BMPR2 expression was variable in the two PAH rat models, while in both experimental models the expression of BMPR2 in the lung vasculature was increased. However, in the human PAH lungs, the expression of pSmad 1/5/8 was downregulated in the lung vasculature of both experimental models. **Conclusion:** BMPR2 receptor expression and downstream signaling is reduced in the lung vasculature of patients with idiopathic and hereditary PAH, which cannot be appreciated when using human whole lung lysates. Despite increased BMPR2 expression in the lung vasculature, the MCT and SuHx rat models did develop PAH and impaired downstream BMPR2-Smad signaling similar to our findings in the human lung.

## 1. Introduction

Mutations in the *BMPR2* gene were the first genetic perturbations implicated in the pathophysiology of pulmonary arterial hypertension (PAH) and are still responsible for most cases of hereditary PAH (hPAH) to date [1,2,3]. PAH patients with a BMPR2 mutation present at a younger age with a more severe phenotype and an increased risk of death [4]. Aside from mutations in the *BMPR2* gene, other genes related to BMPR2 signaling such as *ALK1*, *CAV1*, *ENG*, *SMAD4*, *SMAD8*, *SMAD9*, *BMPR1* and *BMP9* are implicated in hPAH, albeit less frequently [5,6,7,8,9,10]. Furthermore, aberrant BMPR2 signaling has been described in non-hereditary subtypes of PAH, although descriptions of defective BMPR2 expression in human tissue remain relatively scarce [11]. Reduced or absent BMPR2 expression was observed in the lung vasculature of patients with idiopathic PAH (iPAH, then called primary pulmonary hypertension) and hPAH. Decreased levels of BMPR2 were also observed in blood-outgrowth endothelial cells (BOECs) from hPAH and iPAH patients [12,13]. Levels of phosphorylated Smad 1/5/8 (pSmad) were altered in pulmonary artery endothelial cells (PAEC) of iPAH patients compared to controls, indicative of altered BMP signaling [14]. Dewachter et al. showed a lower mRNA expression of *BMPR2* in whole lung lysates from hPAH. In both hPAH and iPAH, *BMPR2* mRNA expression was lower in isolated pulmonary artery smooth muscle cells (PASMCs). The reduced *BMPR2* mRNA expression was not observed in isolated PAEC from iPAH and hPAH patients. In the same study, reduced BMPR2 protein (molecular weight 75kDa) was observed in whole lung lysates of patients with hPAH but not iPAH patients [11]. Together, these findings put aberrant BMPR2 signaling at the center of the pathobiology of many if not most forms of PAH. However, the number of studies assessing BMPR2 expression in the PAH lung remains limited and the methodology used to study BMPR2 expression varied among studies.

It is currently hypothesized that a decrease in BMPR2 signaling leads to a disturbance in the TGF-β/BMP balance favoring the activation of the TGF-β signaling pathway [15]. Increased TGF-β signaling can result in pro-proliferative and anti-apoptotic responses in PAECs and PASMCs, and increased inflammatory cytokine and chemokine production [16,17,18,19]. Although the BMP receptors typically phosphorylate Smad1/5/8 and TGFβ receptors Smad2/3, depending on the cell type and the genetic disorder, TGFβ can induce pSmad 1/5/8, albeit with a different affinity and kinetics when compared to BMP ligands. [20,21,22] Restoring BMPR2 signaling is therefore of interest from a treatment perspective, to stop the progression of the disease. Although, in several translational studies, drugs were tested that were hypothesized to restore the TGF-β/BMP balance and reverse experimentally induced pulmonary hypertension (PH), basic characterization of BMPR2 expression and activity in the most commonly used PH animal models is currently still very limited [23,24,25]. The two most used animal models for PAH preclinical research are the monocrotaline (MCT) and sugen–hypoxia (SuHx) rat models. MCT is an alkaloid derived from the seeds of *Crotalaria spectabilis*. Once injected, MCT is metabolized in the liver into its active form and induces PAEC damage followed by the induction of pulmonary hypertension [26]. The SuHx model depends on a single injection of the vascular endothelial growth factor receptor inhibitor sugen to induce PAEC damage combined with a 4-week hypoxic (10% of oxygen) stimulus [27]. Because both the MCT and SuHx rat models rely on chemically and hypoxia-induced hits for the subsequent development of PAH, in the following weeks rather than decades, it is likely that the BMP signaling pathway in experimentally induced PAH acts differently than observed in the human disease, and might even vary between models. Since in translational research, using the most representative animal model to test new compounds is crucially important, the aim of this study was to describe BMPR2 receptor expression and downstream signaling in the MCT and SuHx rat model for experimentally induced PAH and to compare these findings to altered BMPR2 signaling in the lungs of iPAH and hPAH patients. Additionally, we will compare the expression levels in whole lung lysates, as determined by Western blot, vs. vascular expression only.

## 2. Materials and Methods

### 2.1. Patient Tissue Samples and Animal Experiments

Human lung tissue samples were obtained from hPAH (n = 7) and iPAH (n = 7) patients upon autopsy. Control lung tissue samples (n = 8) were obtained from patients who had died from non-pulmonary causes (cancer, suicide) as previously reported [28]. The usage of samples was approved by the Institutional Review Board of the VU University Medical Center (Approval number: VUMC BUP 2013-5B) and Comite de ´Protection des Personnes (CPP) Ile-de-France VII, Paris (Approval number: 2018-A01252-53). All the samples were formalin fixed and paraffin embedded according to common tissue-processing protocols. Animal experiments were approved by the animal welfare committee of the VU university and were conducted in accordance with the European convention for the protection of vertebrate animals used for experimental and other scientific purposes. Male Wistar rats (MCT model n = 8/control n = 5) and male Sprague Dawley rats (SuHx model n = 8/control n = 5) (Charles River, The Netherlands) were housed in groups of 3–4 under controlled conditions (22 degrees, 12:12 h light/dark cycle). Food and water were available ad libitum.

### 2.2. Study Design

The rats were randomly divided in three groups: control (CON), SuHx and MCT, and the results were compared to both iPAH and hPAH. Control group consisted of a 50/50 mix of Wistar and Sprague Dawley rats. The SuHx protocol was followed as described previously [29]. Briefly, the Sprague Dawley animals were injected with SU5416 (25 mg/kg s.c.), Tocris Bioscience, #3037, Bristol, United Kingdom, dissolved in carboxymethylcellulose (CMC)) and exposed to hypoxia (10%) for four weeks followed by re-exposure to normoxia for 6 weeks. Wister rats received a single dose of monocrotaline (60 mg/kg s.c.) to initiate the development of PAH. The CON group received a single shot of the solvent CMC (SuHx) or sterile saline (MCT). Experiments were terminated 4 weeks post-MCT injection or 6 weeks post hypoxia. The timing of the end-experiments was chosen to ensure the full development of PAH and end-stage pulmonary vascular remodeling [29,30]. The animals were killed via exsanguination and the organs were weighed and processed for analysis.

### 2.3. Right Ventricle Pressure Measurements

Prior to the termination of the experiment, open-chest right ventricle (RV) catheterization was performed under general anesthesia in all animals (3.0% isoflurane, 1:1 O_2_/air mix) as described previously elsewhere [31]. The catheter was inserted through the RV wall. The rats were intubated (Teflon tube, 16 gauge) and attached to a mechanical ventilator (Micro-Ventilator, UNO, Zevenaar, The Netherlands; ventilator settings: breathing frequency, 70 breaths per minute; pressures, 12/0 cm H_2_O; inspiratory/expiratory ratio, 1:1). RV pressures were recorded using a microtip pressure–volume conductance catheter (Millar Instruments, Houston, TX, USA). Analyses were performed when a steady state was reached over an interval of at least 10 s.

### 2.4. Histology and Morphometry

The lungs were weighed and the airways of the right middle lobe were filled with 0.5% low-melt agarose in saline under constant pressure of 25 mmHg and stored in formalin (#4169-30, Klinipath BV, Duiven, The Netherlands). The remaining lobes were stored in liquid nitrogen for future processing. The heart was perfused with tyrode solution, weighed, dissected, snap-frozen in liquid nitrogen and stored in −80 °C. Transversally cut lung sections (4 μm) were stained with Elastica van Gieson (EvG) for the analysis of vascular dimensions. The degree of vascular occlusion was expressed by the percentage of occluded vessels determined on a minimum of 30 vessels per animal per group [31].

### 2.5. Western Blot

RIPA lysis buffer (89900, Thermofisher scientific, Breda, The Netherlands) with protease inhibitors (PMSF, Thermofisher scientific/complete mini protease inhibitor cocktail, Sigma-Aldrich, Zwijndrecht, The Netherlands) were added to the lung tissue and homogenized with a tissue lyser (Qiagen, Venlo, The Netherlands). Protein concentration was quantified by protein assay kit (Thermo Scientific, Pierce Biotechnology, Rockford, IL, USA). The samples (10 µg) were separated on a gradient gel (Bis-Tris 4–12% gel, Life technologies). XCell blot module was used for protein transfer from the gel to an ECL membrane (Hybond ECL Nitrocellulose Membrane, GE Healthcare). Primary antibody for BMPR2 (1:1000, MA5-15827, Thermo scientific, Breda, The Netherlands)/phospho-Smad 1/5/8 (#12656, Cell Signaling, Leiden, The Netherlands)) diluted in phosphate buffered saline with bovine serum albumin (5%) (PBS-A) was incubated overnight at 4 °C. Appropriate secondary antibody (1:4000, Polyclonal rabbit anti-mouse, Z0259, DAKO) diluted in PBS-A was incubated for one hour. The blots were re-incubated with β-actin (1:50000, A3854, Sigma) or GAPDH diluted in PBS for one hour to correct for unequal loading. An internal control composed of a mix of protein supernatants from all groups was used to correct for inter-blot variation.

### 2.6. Immunofluorescence Staining and Quantitative Analysis

After deparaffinization and hydration, epitope retrieval was performed by immersing the slides in antigen unmasking solution (H3300, Vector Laboratories) for 20 min. Blocking steps with 1% bovine serum albumin were performed for 90 min, before incubating the sections with primary antibodies BMPR2 (MA5-15827, Thermo scientific or 612292, BD Biosciences, 1:50) and phospho-Smad 1/5/8 (#12656, Cell Signaling, 1:50) overnight at 4 °C. For the negative controls, the primary antibody was omitted to account for the nonspecific binding of the secondary antibody. Labeling with appropriate secondary antibody conjugated to Cy5 followed for 90 min. Additional overnight staining with pre-conjugated anti-actin α-smooth-muscle-actin—Cy3 (α-SMA, C6198, Sigma), Von Willebrand Factor FITC (VWF, ab8822, Abcam) was performed before a 10 min incubation with 4’6-diamidino-2-phenylindole (DAPI, H-1200, Vector Labs) prior to sealing. Image acquisition was performed on a ZEISS Axiovert 200M Marianas inverted microscope. All the BMPR2 and psmad 1/5/8 images were acquired in a single session. Exposure time for the protein of interest was determined automatically by software (Slidebook 6, Intelligent imaging innovations) once and used for all subsequent slides. The negative control was used to determine and set a low signal threshold. Figures were assembled using an Adobe Illustrator (AI, version 23.03).

BMPR2 expression was determined per high power field (at 400X) and corrected for vessel area by assigning a region of interest (ROI) using FIJI software [32]. ROI was created by manually drawing the vessel circumference subtracted by vessel lumen (if applicable). The average intensity of the BMPR2 signal in ROI was used to quantify the expression per vessel. A minimum of 20 vessels per section were measured. Only the vessels in rats ranging from a 50–100 um diameter were included for analysis.

### 2.7. Statistical Analysis

All the analyses were performed in a blinded fashion. All the data were verified for normal distribution. A *p*-value < 0.05 was considered significant. All the data are presented as the mean ± SEM. The parameters were analyzed by one-way ANOVA with Bonferroni post-hoc testing (GraphPad Prism for Windows 6, San Diego, CA, USA).

## 3. Results

### 3.1. BMPR2 Levels Are not Decreased in Whole Lung Lysates of iPAH and hPAH Patients

**Figure 1:**
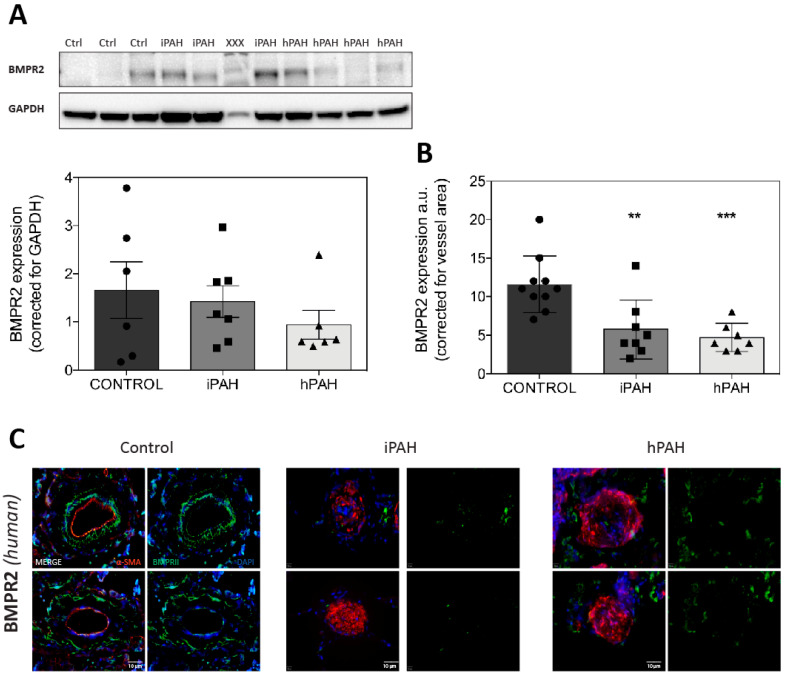
Western blot analysis of BMPR2 protein expression in the whole lung homogenates of idiopathic PAH (iPAH) and hereditary PAH (hPAH). (**A**): BMPR2 expression in the whole lung homogenates. xxx = non-relevant sample. (**B**): BMPR2 expression corrected for the vessel area. (**C**): Typical examples of vessels are shown. Green = BMPR2; red = smooth-muscle-actin (α-SMA); blue = nuclei. ** = *p* < 0.01 / *** = *p* < 0.001.

We first analyzed the protein levels of BMPR2 and pSmad 1/5/8 in whole lung homogenates of samples derived from human PAH patients and controls subjects. Western blot analysis showed that BMPR2 was similar in iPAH and hPAH compared to control lung tissues (Figure 1A). In contrast, semi-quantitative analysis of the expression of BMPR2 in lung vessels by immunofluorescence (IF) revealed the reduced expression of the receptor in the vessels of both iPAH and hPAH patients, compared to the control, regardless of the mutational status (*p* ≤ 0.05) (Figure 1B,C).

### 3.2. Decreased Levels of pSmad 1/5/8 in the Lung Vasculature of iPAH and hPAH Patients

**Figure 2:**
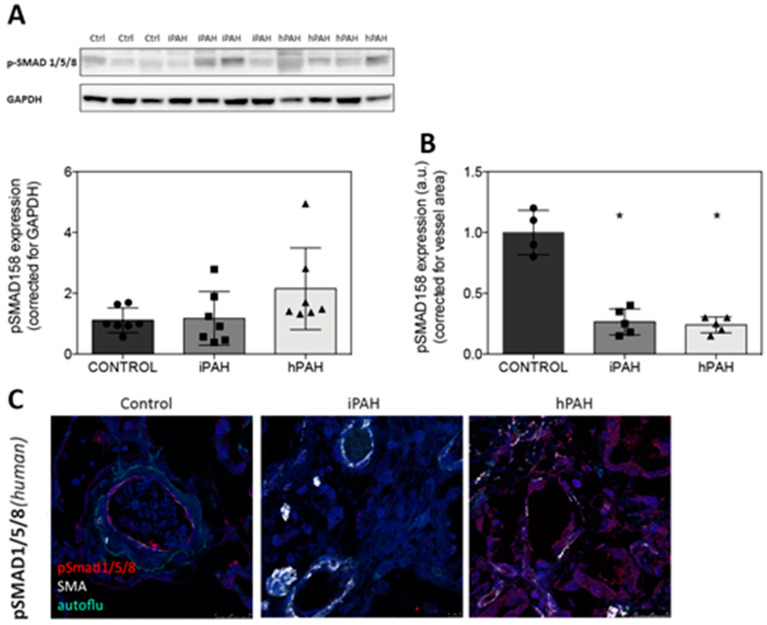
Analysis of Smad 1/5/8/ phosphorylation in the whole lung homogenates. (**A**): pSmad 1/5/8 in the whole lung homogenates. (**B**): pSmad 1/5/8 expression corrected for the vessel area. (**C**): Typical examples of the vessels are shown. Red = pSmad 1/5/8; white = smooth-muscle-actin; blue = nuclei. autoflu=autofluroscence. Scale = 10 μm. * = *p* < 0.05.

Since the BMP Smad1/5/8 are considered to reflect BMP signaling and downstream events, we determined the levels of pSmad1/5/8 in the cell lysates of primary pulmonary micro vascular endothelial cells, following stimulation with BMP9 by Western blot. Like in several cell types, the levels of pSmad1/5/8 are increased, following stimulation with BMP9 in human pulmonary micro vascular endothelial cells (Figure S1). Analyzing the levels of phosphorylated Smad 1/5/8 protein in the whole lung homogenates of the control, iPAH and hPAH groups showed no significant differences (Figure 2A). However, there was a clear reduction in the expression of vascular pSmad 1/5/8 when comparing both iPAH and hPAH to the control vessels (*p* < 0.05) (Figure 2B,C).

### 3.3. Development of PAH in Animal Models

**Figure 3:**
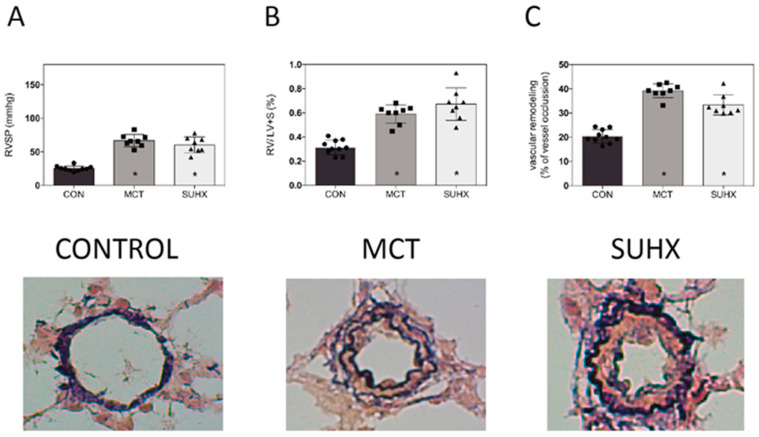
Characteristics of pulmonary arterial hypertension (PAH) animal models: Monocrotaline (MCT) and Sugen hypoxia (SuHx) with (**A**): RVSP = right ventricle systolic pressure, (**B**): Fulton index (RV/LV+S) and (**C**): vascular remodeling. Typical examples of vessel remodeling are shown below (Elastica van Gieson (EvG) staining, 400× magnification). * = *p* < 0.05.

Then, we sought to compare BMPR2-Smad signaling in human PAH with PAH induced in rats. Therefore, we first confirmed the development of PAH in our animal models by the increase in right ventricle systolic pressure (RVSP), the increased RV hypertrophy (RV/LV+S) and the increased vascular remodeling (Figure 3).

### 3.4. BMPR2 Expression Is Increased in MCT and SuHx Lung Vasculature

**Figure 4:**
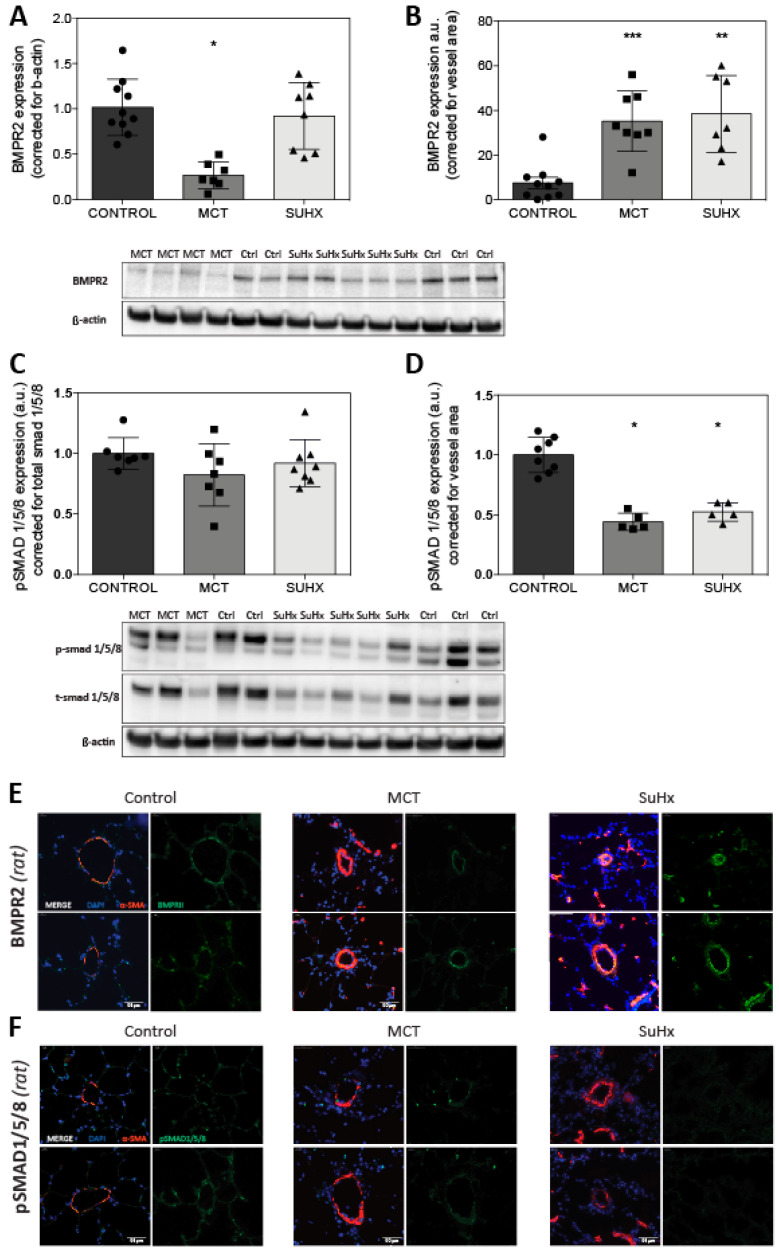
BMPR2 and pSmad 1/5/8 protein expression in the whole lung homogenates vs. immunofluorescence. (**A**–**B**): Analysis of the protein expression by Western blot; and (**C**–**D**): immunofluorescence. (**E**): Typical examples are shown. Green = pSmad 1/5/8 or BMPR2; red = smooth-muscle-actin (α-SMA); blue = nuclei. Scale bar = 50 μm. * = *p* < 0.05/** = *p* < 0.01/*** = *p* < 0.001.

Whole lung BMPR2 protein expression level was reduced in MCT (*p* < 0.05) but did not change in the SuHx model when compared to the control (Figure 4A). Using IF quantification corrected for the vessel area, and we observed a notable increase in the expression of BMPR2 in both the MCT and SuHx lung vasculature (Figure 4B,E). The ratio of pSmad 1/5/8 vs. total Smad 1/5/8, indicating BMPR2 activation, was similar in whole lung homogenates of MCT rats and SuHx rats (Figure 4C). However, the ratio of pSmad 1/5/8 vs. β-actin is reduced in whole lung homogenates of MCT rats (data not shown) indicating that reduced levels of pSmad1/5/8 is due to the reduced total Smad levels and not due to the impaired receptor activation/signaling. IF analysis demonstrated the reduced expression of vascular pSmad 1/5/8 in both PAH models (Figure 4D–F). As we opted to use both Wistar and Sprague Dawley rats in our control group we tested for the statistical difference of whole lung lysate BMPR2 protein expression between both strains. No difference in the BMPR2 protein expression was observed in the separate control group analysis (data not shown).

## 4. Discussion

This study describes the expression levels of BMPR2 and its downstream signaling component pSmad 1/5/8 in lung tissue and more specifically in the lung vasculature of PAH patients as well as in two established animal models of PAH. We confirmed the reduced protein expression of BMPR2 and its downstream signaling component, pSmad 1/5/8, in both hPAH and iPAH, but surprisingly, both the MCT and SuHx rats model for experimentally induced PAH showed an increased vascular expression of BMPR2 in the end stage disease. Downstream BMPR2 activity, as assessed by pSmad 1/5/8 expression, was decreased in the lung vasculature of both PAH models, consistent with the patient situation.

### 4.1. Disturbed BMP Signaling in iPAH and hPAH

The analysis of IF images showed a reduced vascular expression of BMPR2 in both iPAH and hPAH, which is in concordance with a previous report of the reduced expression of BMPR2 in hPAH and iPAH determined by immunohistochemistry (IHC) [13]. While reduced BMPR2 expression in whole lung tissue samples from hPAH but not iPAH was reported in a previous study using Western blot analysis [11], we did not observe a difference in the BMPR2 expression in hPAH/iPAH vs. controls in this study. An explanation for our findings perhaps lies in a different type of mutations that allows for the BMPR2 protein to be formed, but incorrectly expressed or unable to induce Smad 1/5/8 phosphorylation. Another possible explanation is that we detected 115kDa protein of BMPR2 (predicted BMPR2 size is 115kDa) whereas in the other study, we reported the 75kDa protein of BMPR2. Of note, we confirmed the specificity of the BMPR2 antibody by (1) knock-down studies using BMPR2 shRNAs in PAECs and PASMCs, (2) using a different BMPR2 antibody, and (3) using BMP9-stimulated PAEC lysates.

Previous studies showed that missense mutations in the ligand-binding domain by cysteine substitutions impair BMP signaling by mutant receptor mislocalization in the cytosol [33]. Furthermore, as autopsy material was used, it is possible that the cause and time of death may have inferred with BMPR2 signaling in non-vascular cells (e.g., epithelial or macrophage). The phosphorylation of Smad 1/5/8 was decreased in the vasculature of both iPAH and hPAH lungs suggesting that even in the absence of a BMPR2 mutation, BMP signaling is reduced. This observation is in line with another study showing a reduced number of Smad 1/5 positive cells by IHC in the vascular intimal and medial layer of iPAH and hPAH [34]. Richter and colleagues found the expression of phosphorylated Smad 1/5/8 in luminal endothelial cells in plexiform lesions, but pSmad1/5/8 was not present in the core endothelial cells. Unfortunately, the IHC data was not quantified [14].

### 4.2. Reduced BMPR2 and pSmad 1/5/8 Expression in MCT and SuHx Animal Models of PAH

Remarkably, using IF we observed the increased levels of BMPR2 in the lung vasculature of MCT and SuHx rats, while protein levels in the whole lung lysates were only reduced in the MCT model. Previously, Ramos et al [35]. reported reduced levels of BMPR2 protein by Western blot analysis using whole lung lysates 14 days after MCT injection. Others have described a brief initial increase in BMPR2 protein expression two days after MCT injection, with a subsequent decrease in BMPR2 protein levels after seven and twenty-one days [36,37,38] in whole lung homogenates by Western blot analysis. Impaired BMP signal transduction was confirmed in our study by reduced pSmad 1/5/8 expression in both MCT and SuHx. A recent study assessing the increase in BMPR2 signaling upon BMP9 stimulation observed the reduced levels of BMPR2 and pSmad 1/5/8 in the MCT model. For SuHx, only a reduction in the expression of pSmad 1/5/8 was reported [39]. Other additional studies have also reported decreases in protein expression of pSmad 1/5/8 [35,40]. In contrast to these and our studies, one study reported augmented Smad1 signaling [35]. One difference in the methodological setup between this study and ours is the time post-MCT injection when the analysis was performed (14 vs. 28 days post-MCT). Since BMPR2 expression varies over the course of disease progression in the MCT model, the time difference is likely to exp--lain the difference in Smad1 signaling [37]. Our study opted for prolonged disease development before the analysis of the experimental PAH models, as at that time-point, it more closely resembles human end-stage disease. One explanation for the seemingly normal vascular BMPR2 expression in the SuHx model might be the role of vascular endothelial growth factor receptor 3 (VEGFR3). The blocking of VEGFR3 by sugen contributes to the vascular remodeling observed in the SuHx model, but recently VEGFR3 has also been linked to BMPR2 and PAH [41,42]. Hwangbo et al. propose a VEGFR3–BMPR2 interaction that is crucial for receptor endocytosis and the subsequent induction of the phosphorylation of Smads [43]. The inhibition of endocytosis by blocking VEGFR3 by sugen might therefore result in an increased BMPR2 vascular expression observed in PAH.

Analyzing vessels in more detail showed that in both MCT and SuHx animals, pSmad 1/5/8 is reduced while BMPR2 expression is increased. This increase in BMPR2 levels might be a feedback mechanism to restore endogenous BMP signaling and prevent disease progression, a process that has failed in the end-stage lung tissue of iPAH patients. It could also imply differences in BMPR2 and/or the type I receptor kinase activity [15], or the presence of inhibitors preventing BMPs binding to the receptor [15,44]. While our study was able to distinguish between the vascular vs. non-vascular expression of BMP signaling, it will be worthwhile to differentiate between the different vascular cell types. Even within the population of endothelial cells, the expression of phosphorylated Smad 1/5/8 was shown to differ upon location within a plexiform lesion [14]. In addition, the characterization of BMP signaling throughout the disease development might give more insight into the role of BMP signaling in the vascular remodeling in PAH. Perros et al. recently published their findings from a novel BMPR2 mutant rat model aimed to specifically model hPAH. These BMPR2 mutated rats spontaneously developed PAH between six and twelve months of age. Whole lung BMPR2 and pSmad 1/5/8 protein expression was lowered by fifty percent as measured by Western blot analysis [45]. Analyzing the lung vascular BMPR2 and pSmad 1/5/8 expression in this novel model would inform us if the expression is indeed similar to human disease.

### 4.3. Interventions Aimed to Restore the TGF-β/BMP Balance in Experimental PAH

The inhibition of the lysosomal degradation of BMPR2 by chloroquine prevented the progression of PAH in the monocrotaline (MCT) model [46,47]. The restoration of downregulated BMPR2 signaling via the targeted gene delivery of *BMPR2* revealed the therapeutic potential in the MCT model [36]. Sildenafil, a phosphodiesterase type 5 inhibitor currently registered for the treatment of PAH, was demonstrated to restore MCT-induced PH in association with increased whole lung pSmad 1/5/8 expression [48]. Using laser dissection microscopy, Mcmurtry et al. showed the decreased BMPR2 mRNA expression in the MCT lung vasculature. Additionally, they showed a gradient in lung vascular BMPR2 mRNA expression with distal vessels having the highest expression levels compared to mid or proximal vessels. Gene therapy with BMPR2 distributed BMPR2 to resistance pulmonary arteries but did not ameliorate PAH in the MCT model [38]. Furthermore, in a study assessing the effect of a selective TGF-β ligand trap, decreased levels of BMPR2 mRNA in SuHx and MCT lungs were unaffected by this treatment despite the effective reversal of PH [49]. Post or propter activation of BMPR2, FK506 (known as tacrolimus) reverse established PAH in the SuHx and MCT models [23,50]. In another study, the selective enhancement of BMPR2 signaling using BMP9 was shown to reverse disease in the MCT and SuHx models. In this study, whole lung pSmad 1/5 was reduced before treatment in both the MCT and SuHx model, while only in the MCT model was the lower whole lung BMPR2 protein expression was reported [39]. A multitude of other studies, investigating the compounds targeting BMPR2 signaling regulation, protein processing regulation, translational regulation, transcriptional regulation and genetic based therapies were performed or are currently ongoing [51,52,53,54].

The aim of this study was to give insights in the translational capability of two animal models commonly used to study new treatment modalities for PAH. Focusing on the BMPR2 signaling pathway as the main driver of the disease, both animal models only partly recapitulated the human situation. This study reconfirmed and added to the still limited body of evidence describing decreased vascular BMPR2 expression in PAH. Our contrasting findings of increased BMPR2 observed in the SuHx and MCT lung vasculature emphasizes the inadequacy of using whole lung lysates. We propose for future studies to not use whole lung lysates to determine BMPR2 levels, but more suitable techniques such as IF or IHC, which will allow to take into account the spatial localization of BMPR2 expression. Since the expression levels of BMPR2 are different in both MCT and SuHx models compared to hPAH or iPAH, this should be taken into consideration when performing preclinical studies using a candidate compound to target BMPR2 or its downstream signaling component.

**Figure 5:**
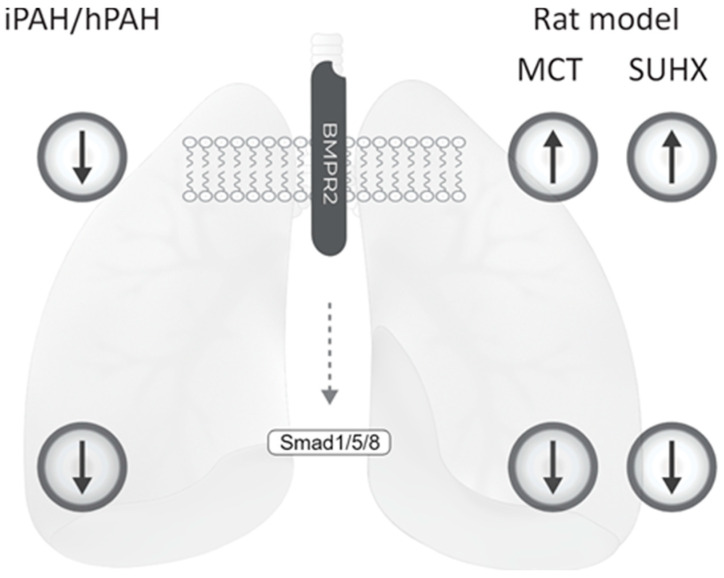
Schematic overviews summarizing the imaging results of this study. While BMPR2 protein expression is decreased in PAH along with decreased pSMAD 1/5/8 signaling, animal BMPR2 expression is increased in the lung vasculature.

In conclusion, our findings confirm decreased BMPR2 receptor expression and downstream signaling activity in the lung vasculature of patients with idiopathic and hereditary PAH. At the same time, our study indicates that whole lung lysates are not representative of vascular BMPR2 and pSmad 1/5/8 expression in the lung. Finally, despite an increased BMPR2 expression in the lung vasculature, PAH developed in the MCT and SuHx rat models and the downstream BMPR2 signaling activity resembled that of human PAH (Figure 5).

## Supplementary Materials

The following are available online at https://www.mdpi.com/2073-4409/9/6/1422/s1, Figure S1: BMP9 induces pSmad1/5/8 levels in microvascular endothelial cells.
