# Supplementary material for: The BMP Receptor 2 in Pulmonary Arterial Hypertension: When and Where the Animal Model Matches the Patient
Source: Cells. 2020 Jun 8;9(6):1422. doi: 10.3390/cells9061422 (PMC7348993; doi:10.3390/cells9061422)
Supplement: Supplementary file 1 [file cells-09-01422-s001.pdf]

Figure S1

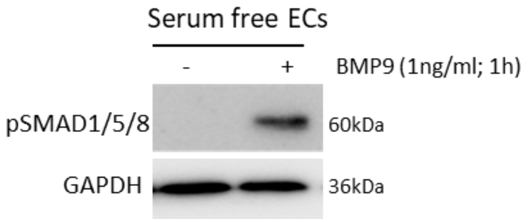

Fig S1: *pSmad1/5/8 levels in serum starved microvascular endothelial cells treated with or without BMP9 (1ng/ml) for 1h, indicating BMP9 can induce pSmad1/5/8 in these cells.*
